# Thinking Theoretically in Nursing Research—Positionality and Reflexivity in an Interpretative Phenomenological Analysis (IPA) Study

**DOI:** 10.1111/nin.12684

**Published:** 2024-11-11

**Authors:** Iyore. M. Ugiagbe, Helen. T. Allan, Michael Traynor, Linda Collins

**Affiliations:** ^1^ Department of Adult Nursing Faculty of Health, Social Care & Education, The Williams Building, The Burroughs, Middlesex University London UK; ^2^ Health and Human Sciences Southeastern Louisiana University Hammond Louisiana USA

**Keywords:** ethnicity, integration, Internationally Educated Nurses, Interpretative Phenomenological Analysis, positionality, recruitment, reflexivity

## Abstract

This paper explores the application of positionality and reflexivity drawing on the experience of a British Minority Ethnic (BME) group senior nurse researching nurses with the same ethnic heritage in an IPA study. It explores how using IPA informed reflexivity and positionality as a researcher who shared the same ethnicity with the research participants. The IPA study allowed for the exploration of Internationally Educated Nurses' (IENs) perspectives on their integration into British healthcare and their navigation of career progression. The central aims of an IPA study are to understand the participant's world, its description, the development of a clear, and open interpretative analysis with a descriptive focus on the social, cultural and theoretical context, and the participant's sense‐making of their lived experience. In this paper, we discuss how the lead researcher employed reflexivity, stated his intentionality and positionality in the conduct of the IPA study. This paper discusses some examples of the effects of positionality and reflexivity in the conduct of research by researchers of different racial background, and explicate the influence of personal and professional experiences of a researcher in using reflexivity and positionality to ensure cross‐cultural validity and reliability of an IPA research. This paper concludes that appropriate use of reflexivity and positionality in an IPA study may recognise the personal and professional influence of a researcher's experiences on the research process, including their ethnicity.

## 1 Background

‘The dancer often believes that he is dancing well, but it is the objective onlookers that can judge correctly if he is a good dancer.’ (A Benin (Edo); Nigerian parable)

This paper explores the use of positionality and reflexivity in an Interpretative Phenomenological Analysis (IPA) study. We discuss how the use of reflexivity and positionality informed by IPA methodology may recognise the personal and professional influence of a researcher's experiences on the research process, including their ethnicity. We draw on the experience of conducting a qualitative study of Internationally Educated Nurses' (IENs) with Nigerian heritage integration into UK healthcare in the London region. The IPA qualitative study also explored how ethnic minority nurses attain and thrive in senior positions in British healthcare. It contributes to the generation of theoretical, practice and policy‐influencing knowledge to promote understanding the ‘how’ of workforce race equality (WRES 2021) in the British healthcare system—the National Health Service [NHS) (Ugiagbe 2022).

The UK Nursing and Midwifery Council (NMC) registered more than 52,000 new nurses in 2022/23, and 25,006 of these professionals were IENs (NMC 2023). The sustained increase in the number of IENs on the NMC register underlines the need for more research on integrating IENs into British healthcare. Despite the continuing and increasing number of IENs in UK healthcare over the years, ethnic minority nurses, especially people of African descent, continue to experience discrimination more than any other groups in employment, promotion, and training [Archibong and Darr 2010; Kline 2014; Likupe et al. 2014; West, Nayar, and Taskila, 2017, Ugiagbe 2022]. Nurses with Nigerian ancestry represent the most significant number of Africans in the NHS (Barker 2023). This in part justified the IPA study of IENs to understand the lived experiences of Nigerian nurses in integrating and attaining promotion in the face of documented racial discrimination.

The IPA approach offered the best opportunity to ‘*understand the innermost deliberation of the "lived experiences" of research participants’* (Alase 2017, 10). The central aims of an IPA study are to understand the participant's world and its description as well as the development of a clear and open interpretative analysis with a descriptive focus on the social, cultural and theoretical context in addition to the participant's sense‐making of their lived experience (Larkin, Watts, and Clifton 2006). According to Allan and Westwood (2015), a researcher's background may shape the research, and a researcher's use of reflexivity and declaration of positionality promotes honesty and openness in interpretations of the phenomenon under study (Ugiagbe 2022). In this paper, we discuss how the lead researcher drew on his experience as a British Minority Ethnic (BME) group senior nurse researching nurses with the same ethnic heritage, to develop his use of reflexivity and positionality in an IPA methodology. Reflexivity and positionality were enhanced through the sharing of ethnicity and reflexively using ethnicity as a lens in the data analysis. We discuss examples of the effects of positionality and reflexivity in the conduct of research by researchers of different racial background, and explicate the influence of personal and professional experiences of a researcher in using reflexivity and positionality to promote cultural competence and congruency to ensure cross‐cultural validity and reliability of an IPA research.

## 2 Theoretical Framework

The researcher in an IPA study needs a dedication to understanding the participant's ‘lived’ experience, and this requires interpretative work on the researcher's part (Smith 2004). It offers a systematic approach to idiographic inquiry (Smith and Osborn 2007), in which the researcher analyses individual cases in great detail and uses extracts from the participants' narratives to offer explanations (Ugiagbe 2022) about the phenomenon. IPA focuses on the lived experience of a social phenomenon (Giorgi 2009) to illuminate and understand (Groenewald 2004) that phenomenon through a direct description and interpretation of the ‘what’ and the ‘how’ of the individual's experience. The study of integrating IENS with Nigerian heritage in the British healthcare system explored the lived experiences and social processes of IENs' experiences of integration and sought to contribute to developing a theory of the necessary social process of integrating IENs into the British healthcare and society (Ugiagbe 2022).

The researcher's role in IPA is to determine the meaning of participants' experiences through understanding and interpretation of their lived experiences (Ugiagbe 2022). Smith, Flower and Larkin (2012, 3) refer to this interpretation as double hermeneutic because the ‘*researcher is trying to make sense of the participant trying to make sense of what is happening to them.*’ The double hermeneutic process in IPA enables a better position to approach the topic and allows a researcher to use reflexivity to define positionality, honesty and openness in interpretations of the phenomenon under study (Ugiagbe 2022). The researcher needs to be conscious of a third hermeneutic level (Smith, Flower, and Larkin 2022, 182), where the researcher should always imagine the reader's interpretations being ‘*positioned as attempting to make sense of the researcher trying to make sense of the participant's experience*.’ This is the point where the Benin (Nigerian) adage ‘*the dancer often believes that he is dancing well, but it is the objective onlookers that can judge correctly if he is a good dancer*’ becomes significant in conducting an IPA study and emphasises the significance of applying reflexivity at every stage of the study.

In agreement with some other researchers on reflexivity, in this paper we do not consider reflexivity and reflection to be mutually exclusive (Karcher, McCuaig, and King‐Hill 2024, Lazard and McAvoy 2020; Shaw 2010). Reflexivity is ‘a continuing mode of self‐analysis’ (Callaway 1992, 33) and explains how the researcher's experience has or has not influenced the stages of the research process. It requires the researcher to continuously self‐critique and engage in self‐appraisal (Koch and Harrington 1998). It examines the researcher's position, self‐scrutiny, and self‐conscious awareness of their relationship with an ‘other’ (Bourke 2014). Reflexivity is ‘*…the process of a continual internal dialogue and critical self‐evaluation of researcher's positionality as well as active acknowledgement and explicit recognition that this position may affect the research process and outcome*’ (Berger 2015, 220). Engaging in the use of reflexivity enables a researcher to apply the research's ‘etic’ and ‘emic’ levels in sustaining a drive to analyse the data of the study. Reflexivity is a ‘*detailed evaluation of the self*’ (Shaw 2010, 234). It enables researchers to acknowledge that their actions and decisions will inevitably impact the meaning and context of the experience under investigation (Horsburgh 2003, 209). The IPA researcher needs to become fully aware of the actual and potential factors that may impact the way the data may be approached and must be clear about the process of engagement with the data as well as provide an audit trail to increase the level of trustworthiness of the research (Rodham, Fox, and Doran 2014).

Reflexivity provides an avenue for improving self‐awareness in any study. This process lets the researcher elucidate positionality and how emerging views concerning the individuals' values, interests, and beliefs may shape the research objectives (Cousin 2009). Karcher, McCuaig and King‐Hill (2024), and Mann (2016) promote the use of research journal as a primary tool to encourage reflexivity in studies such as IPA. Research journaling may remind a researcher of meaningful discussions and issues and make the event descriptions complete and easy to remember (Ugiagbe 2022). In addition to maintaining a journal such as an e‐journal, engaging in a reflexive interview, data analysis, and discussion are examples of ensuring reflexivity in an IPA study. Other tools to encourage reflexivity in an IPA study include a review of comments from the research supervisory team, discussions in conferences, and reflective discussion of the topic of study with professional colleagues.

Positionality contextualises the researcher and research environment in defining boundaries of the research (Jafar 2018). It is recognising and declaring a researcher's position in a study. This enables the reader to make an informed decision as to what extent the researcher's positionality and other personal experiences may impact the conduct and interpretation of the research findings (Ugiagbe 2022). Smith, Flower and Larkin (2022, 182) refer to this as the researchers being conscious of the third hermeneutic level in IPA study in which the researcher always imagines the reader's interpretations being ‘*positioned as attempting to make sense of the researcher trying to make sense of the participant's experience.*’ In the study of the integration of Nigerian IENs into the British healthcare system, drawing on his own experience of education and work in Nigeria and the UK, the lead researcher began to see how the experiences of the Nigerian nurses born in the UK and those born in Nigeria were subtly different and how this in turn shaped their integration and promotion in the NHS. The lead researcher became aware of the additional challenges and opportunities posed by: i) sharing the same ethnicity as the research participants, i.e. Nigerian heritage, ii) having also been a senior manager in the NHS and having experienced direct discrimination, iii) speaking English as a second language and as the language used in conducting the study. In this example, the lead researcher used reflexivity to examine his position, the role of possible bias in his decisions, and the processes used to reduce bias in the research (Hannes 2011). The lead researcher had assumed that the research participants were born in Nigeria. Significantly, the interview revealed that 30 percent of the participants were in fact born in the UK but spent their childhood until their young adult years in Nigeria before returning to live in the UK where they now have dual citizenship. The lead researcher was richly educated by the experiences shared by these participants. By thinking and acting reflexively, a researcher makes his positionality explicit to himself and/or others, influenced in varying degrees by the researcher's ethnicity, age, life experiences, social identity, role and personality (Jootun, McGhee, and Marland 2009), and the degree of influence of each factor vary depending on relevance and the research objectives (Ugiagbe 2022).

The lead researcher in the IEN study used emancipatory theories of Critical Race Theory (CRT), Intersectionality Theory (IT) and Postcolonial Theory (PCT) to explore existing social, economic and political dimensions and discourse on discrimination; and to provide a comprehensive understanding to challenge the current integration discourse and status quo in IENs employment and integration in UK healthcare. Reflexively, the lead researcher's use of emancipatory theories offered a solid basis to critique the philosophy and practice of IENs recruitment and integration into UK healthcare, and promote thinking to analyse the roots of IENs recruitment and integration beyond the factors evident at the surface level (Ugiagbe 2022).

## 3 Method of Reflexivity

Reflexivity may be used as ‘*the primary methodological instrument for an inquiry e.g. in autoethnography‐ autobiography and narrative inquiry*’ (Dowling 2013, 9), and reflexivity may be about the researcher's interpretation of the context of the phenomenon. The researcher's perspective, worldview, and criticality are essential in the researcher's attitude and or practice in reflexivity (Ugiagbe 2022). The main plot of this paper is to discusses how the lead researcher employed reflexivity, stated his intentionality and positionality in the conduct of the IEN study using IPA. In this paper, the authors compare examples of the effects of positionality and reflexivity in the conduct of research by different racial groups, appropriate recognition of personal and professional influence of a researcher's experiences on the research process and how reflexivity and positionality facilitate the researcher's application of cultural competence and congruency in shaping the research process to ensure cross‐cultural validity and reliability of the research. The IEN study focussed on BME nurses of Nigerian descent employed in the British healthcare for between 5 and 30 years. The researcher's efforts to recruit from a population of senior nurses (band 8 and above) of Nigerian descent proved difficult because ‘*despite London trusts generally having the highest proportion of BME staff in the country, representation at senior band is very low*’ (WRES 2019, 16). Almost all the nurses known to the researcher had been out of their senior managerial roles or some retired prematurely taking advantage of the option available to them during organisational restructure exercises. In line with the reasoning of Kinsella (2010), that phronesis (wise action) needs to be used in professional practice to complement episteme (scientific knowledge) and techne (pragmatic knowledge), the lead researcher modified sampling to recruit senior nurses of Nigerian descent on pay band 7 and above. Reflecting on the reasons that limits the availability of BME in the more senior position, the decision to reviewing the sample to include pay band 7 nurses, which is the gateway to getting to pay band 8 and above, positively impacted the importance of the IEN study. Purposive and snowballing approach was used to recruit ten senior NMC registered nurses working at pay band 7 or above. Data collection used open‐ended questions in recorded semi‐structured interviews conducted in the participant's preferred venue, and in a conducive and relaxed atmosphere to enable the participants to tell their own stories or lived experience. The semi‐structured interview was supported by interview prompts. To maintain and offer sound interpretation, the lead researcher as a member of an ethnic minority group who was researching his own community group, employed reflexivity, stated his intentionality and positionality in the discussion, data collection, and analysis. At times during the IENs study, the lead researcher's positioning gave rise to different insights to the perspectives of one of the white research supervisors. The principal supervisor (white) has done several studies on international nurses over the years. Whereas, the principal supervisor was quick to understand the relevance of the IENs research, the initial second supervisor who was also white struggled to agree to the relevance of the study. The lead researcher had to engage in a determined explanation to explain and convince the supervisory team on the significance of the IEN study before the initial second supervisor supported the idea. Reflexively, the second supervisor's initial reluctance may have been because there was little or no literature on long term integration of BME nurses in the British healthcare. Most of the studies on the BME nurses have always been on the problems BME nurses encountered rather than the solutions to the issues and recognition of what works (Ugiagbe 2022). The lead researcher wrote down some ideas as short notes or memos, that helped in analysis and helped discover the lead researcher's otherwise hidden knowledge (Ugiagbe 2022). The lead researcher used e‐journal before and after interviews to clarify his knowledge, and engaged in critical reflection. In addition to maintaining an e‐journal, the lead researcher engaged in reflexive interview, discussion and review of comments from the supervisory team, discussions in conferences and with professional colleagues to help the lead researcher in reflexivity.

## 4 Reflexivity in Analysis

In any IPA study, it is imperative for the researcher to maintain positionality and use reflexivity in each stage of the research. In thinking reflexively, the lead researcher constantly monitored personal beliefs, bias, and research experience in keeping the e‐journal, and in engaging supervisory discussion sessions. The lead researcher used the double hermeneutic concept in IPA to become aware of preconceptions and potential influences on the research. In transcribing the recorded interview, listening to the pauses and recollecting the non‐verbal utterances brought alive empathetic feelings and deep reflection during initial interpretation of the statement or comment by the participant of their line of thought (Ugiagbe 2022). Reflecting on the impressions of the interviewer –interviewee interaction using the record from the researcher's e‐journal, generated additional sources of data that became useful in ‘*contextualization and development*’ of the findings (Smith, Flower, and Larkin 2012, 73). For example, one of the interviewees in the IEN study in discussing the reasons for lack of integration of some ethnic minorities in the UK healthcare and the larger society, argued that there was mistrust, prejudice, and lack of knowledge about human existence and interdependence in the society. The interviewee in explaining the need for tolerance, understanding and appreciation of diversity of the different ethnicity in the UK stated:

‘Yes, yes. I think it is just colour threats. They fail to realise the need for integration. They need to know (pointing to an advert in the Metro newspaper which reads) –

‘We are not an Island. We are a Colombian coffee‐drinking, American movie watching, Swedish Flat‐pack assembling, Korean tablet tapping, Belgian striker supporting, Dutch beer cheers‐ing, Tikka Masala Eating, Wonderful Little lump of land in the middle of the seas. We are part of something far, far bigger’ (HSBC UK, 2018)” (Page 22, line 208) to emphasise the interdependence between people of different nations and the need to accept one another in promoting integration.

Reflexivity helped the lead researcher in the analysis to objectively offer a sound interpretation of the findings in the IENs study. In IPA, the researcher does not bracket preconceptions or theories but uses reflexivity to be fully aware of his meanings of the real world and his existence in the world of study (Johnson 2000; Lowes and Prowse 2001). The researcher's personal and epistemological reflexivity advances more significant opportunity to understand the phenomenon under study and the research process. This supports the perspective of Kinsella (2010) in the use of phronesis (wise action) in areas where technical solution requiring episteme (scientific knowledge) and techne (pragmatic knowledge) in professional life may not be suitable in resolution of the issue at hand.

McConnell‐Henry, Chapman and Francis (2009) state that the researcher's previous understanding and knowledge help interpretation, and they maintain that bracketing has no place in interpretive phenomenology, but Finlay (2008) disagrees and argues that bracketing or reduction involves the researcher acknowledging his influences and possible bias in interpretative phenomenology. The lead researcher in the IEN study used reflexivity to encourage participants to share their reflections on the phenomenon under study. The lead researcher was consistently aware of his positionality, and became immersed in the participants' level of trust in sharing their experiences with him as an ethnic minority researcher. The lead researcher struggled not to share his personal experiences to validate the experiences or encourage the spirit in the participants' quest to remain successful in their professional pursuit.

In transcribing the interviews, the lead researcher paid attention to the tone and re‐visualised the bodily expression during the interviews. Reflecting on the interview process and analysing the interview guided the lead researcher not to stray too far from the research questions, as this may have produced very emotional data with little or no relevance to the study (Ugiagbe 2022). Moving between the idiographic component to establish convergence and divergence, commonality and individuality within the data generated was challenging for the lead researcher. The iterative engagement with the analytic process and keeping ideographically close to the research participants promoted the retention of idiographic voice to maintain a joint claim for the participants in the IPA study (Ugiagbe 2022).

In interpreting data and writing the analysis of the interviews, the lead researcher searched for commonality across the participants' experiences, and ‘*choose some atypical extracts to illustrate contradiction and complexity*’ (Smith, Flower, and Larkin 2012, 116). In emphasising the idiographic element in individuals’ interpretation of their lived experiences because of their differences in expression or description of their lived experiences, the lead researcher used different length of quotes in the analysis. To recouple themes and relate to the fundamental analysis to develop across the different sections of the IPA study (Ugiagbe 2022), the lead researcher engaged in some iterative accounts to be more interpretative of the respondents' lived experiences.

## 5 Using Reflexivity and Positionality in Findings

The application of positionality and reflexivity in the exploration of the IENs' perspectives on their integration and navigation of career progression in British healthcare while drawing on the researcher's experience as a British Minority Ethnic (BME) group senior nurse of the same ethnic heritage was challenging and revealing for the lead researcher. The IEN study, offered insight and further understanding of the post‐transition phases and the long‐term integration of the IEN with Nigerian heritage into British healthcare. Findings from the IEN study indicated that integration in the UK and UK healthcare services was a complex phenomenon shaped by immigration processes, employers' practices, social capital, discrimination, mentoring and personal characteristics such as education, resilience, motivation and personal values (Ugiagbe 2022). The findings also suggested that most of the highly successful IENs of Nigerian heritage in UK healthcare tend to be those who had tertiary education in Nigeria before becoming registered nurses in the UK.

A researcher's personal experiences, interests, negative or positive organisational outcomes, challenges in areas of work or issues that require proof or lack answer (Roberts 2010; Lipowski 2008) has influence on research because *‘all research represents a political enterprise with significant implications* (Streubert‐Speziale and Carpenter 2007, 19). However, disclosing positionality and experience in reflexivity increase the quality of research, trustworthiness, rigour and accountability (Mann 2016). In a scoping review on reflexivity, Karcher, McCuaig and King‐Hill (2024) stated that many researchers including early career researchers who work on emotional topics feel unprepared and received little or no training or support for reflexivity practices. As a graduate student ethnic minority researcher, researching IEN integration into UK healthcare posed a different challenge for the lead researcher from that experienced by accomplished white researchers, such as described in Allan (2022) “*Reflections on whiteness: Racialised identities in nursing*” and Bourke (2014).

In thinking theoretically, the lead researcher in the IEN study had to declare positionality, and used reflexivity in understanding the findings from the lived experience. The findings from the IENs study, appear to be what were significant to the participants' recollection of their lived experience in the UK (Ugiagbe 2022). As stated by the lead researcher, the analysis of the data was directed to answer the research question and therefore every area of the respondents lived experience may not have been covered since the thrust of the experiences were to answer the research question. Thinking theoretically, the resulting analysis was probably one possible account of the lived experiences of the participant's integration experience (Ugiagbe 2022).

The lead researcher's BME background, sharing same ethnic heritage and first‐hand experience of discrimination in the NHS helped to consistently think and apply ethical principles in discussing the findings, which also explained the complexities of discrimination in the clinical environment and the wider society. The difference in how the participants expressed their experiences was a significant finding, and the lead researcher had to maintain positionality by engaging ‘etic’ and ‘emic’ levels to analyse data and the findings of the study (Ugiagbe 2022). Consequently, monitoring personal beliefs, bias, and research experiences, and constant awareness of preconceptions and potential influences on the research, were measures employed by the lead researcher to think theoretically and to maintain positionality and reflexivity.

According to Hall (1990, 258), ‘*practices of representation always implicate the positions from which we speak or write—the positions of enunciation*’ and it is in stating a researcher's positionality that there is a balance in the issue of objectivism and subjectivism in the research. Engaging positionality at the ‘etic’ and ‘emic’ level may have different impact for the researcher and the research. For example, from the findings of previous research which suggest that human beings tend to gravitate toward those with whom they share some level of commonality, Bourke (2014, 2), who described himself as a ‘*White, heterosexual, cisgender male*,’ assumed a positionality based on race in conducting a focus group interview, and his finding was contrary to his expectation. According to Bourke (2014), ‘*Students of color were much more open to discussing issues of race with me, while White students were what can best be described at reticent*.’ Reflexively, in the IEN research, the lead researcher discovered that an assumption at the start of the study that the senior nurses were born in Nigeria was proved wrong. The data revealed that 30 percent of the senior nurses were born in the UK but spent their childhood to young adult years in Nigeria before returning to live in the UK. These senior nurses identified as Nigerians in social relationship, declared that they were Nigerians when recruiting participants but maintained ‘Britishness’ during the interview sessions. The data also revealed that all the respondents now have dual citizenship of Nigerian and British (Ugiagbe 2022).

By reflexivity, the lead researcher maintained a professional stand to decipher the difference in the display of emotions and rationalisation of lived experiences between the nurses born in the UK and those born in Nigeria. The lead researcher was immersed in the participants' level of trust in sharing their experiences. As an ethnic minority researcher, there was resonance between the respondents' lived experience and the lead researcher's life experiences in a number of areas but using reflexivity and using the double hermeneutic concept of IPA assisted the lead researcher not to share personal experiences to validate the respondent's experiences or support or encourage their spirit in their quest to remain successful in their professional pursuit (Ugiagbe 2022).

Using reflexivity helped in the lead researcher's thinking when listening and interpreting the participants use of words, the level and depth of the general issues that the respondents discussed. The lead researcher had to constantly use reflexivity to shield his emotion and to provide a listening ear during the interview session. In thinking theoretically, reflexivity and declaring positionality guided the lead researcher from producing very emotional data with little or no relevance to the study (Ugiagbe 2022). Engaging in reflexivity helped the lead researcher to stay on track and to answer the research questions.

In analysis of the data, reflexivity helped the lead researcher to engage iteratively with the analytic process and retain the idiographic voice of respondents whilst maintaining a joint claim for the participants in the study (Ugiagbe 2022). The lead researcher ‘*choose some atypical extracts to illustrate contradiction and complexity*’ (Smith, Flower, and Larkin 2012, 116) to emphasise the idiographic element in individuals’ interpretation of their lived experiences.

## 6 Discussion: Positionality and Reflexivity In IPA

IPA is an iterative and inductive cycle that draws on innovative strategies (Smith and Osborn 2007). The lead researcher used an iterative and inductive process of de‐contextualization and re‐contextualization to analyse the data captured. The lead researcher then used the hermeneutic circle to prompt questions to understand the essence of ‘being’ and to discover the true meaning of the phenomenon under study. Through dialogue and openness between the lead researcher and the participants, the lead researcher became part of the historical, social, and political world of the participants (Ugiagbe 2022), and gave better understanding and discussion, resulting in ‘*mutual construction of the reality and identification of ‘the meaning or essence of the experience’* (Tuohy et al. 2013).

Dowling (2013) advocates that there are generally two principal types of reflexivity: personal and epistemological. Personal reflexivity is described as self‐awareness (Giddens 1976) and mirrors reflection as a learning tool. In epistemological reflexivity, a researcher can reflect on how the research question shaped or defined and limited the findings and other ways the research may have been conducted differently. Four forms of reflectivity are associated with the issue of validity in research (Koch and Harrington 1998). The first form of reflexivity is the reflexivity aimed at sustaining objectivity and reflecting a strong positivist influence, e.g., using bracketing (reflective journal); secondly, the epistemological reflexivity. In epistemological reflexivity, significant consideration includes self‐critique and an openness to the possibility of more than one interpretation of a research product (Ugiagbe 2022). This form of reflexivity has a strong personal nature and resonates with Gadamer (1997) view that one must first understand oneself to understand another. Thirdly, reflexivity from a critical standpoint–examining the political and social constructions influencing the research process and fourthly, reflexivity from an experiential standpoint as in feminist perspectives (Dowling 2006). The use of reflexivity in the IPA study of integrating IENS with Nigerian heritage in the British healthcare system required a keen appreciation of the challenges posed by the review of the literature and the lead researcher's nursing professional experience as senior ethnic minority nurse. It was necessary to disclose positionality and engage in reflexivity experience (Mann 2016) in the lead researcher's diary, in research supervisory sessions, in interviews and analysis and findings to enhance the quality, trustworthiness, rigour and accountability of the research.

Reflexivity starts by identifying preconceptions brought into the project by the researcher, representing previous personal and professional experiences, pre‐study beliefs about how things are and what is to be investigated, motivation and qualifications for exploration of the field, and perspectives and theoretical foundations related to education and interests (Malterud 2001, 484).

In the IPA study of integrating IENS with Nigerian heritage in the British healthcare system, Malterud (2001) reasoning influenced the use of reflexivity by the lead researcher at every stage of the IENS study. The lead researcher engaged reflexivity from the stage of writing the research proposal through to the refining and transfer stage of the research programme, and throughout the conduct of the research. Reflexivity resulted in awakened understanding and consciousness that ‘*the critical exercise of cultural power and normalisation*” has serious effect on “*the ways in which black people, black experiences, were positioned and subject‐ed in the dominant regimes of representation*’ (Hall 1990, 260) in the dominant literature. Reflexivity may be a method to reflect on one's positionality and may serve as a control and measurement tool in a study. Allan (2022) and Bourke (2014) provide excellent examples of reflexivity in practice, and the use of reflexivity in discussing positionality in studies on IENs and ethnic minority students as a white researcher. Bourke (2014, 1) stated that ‘*throughout my preparations to conduct this research, from the formulation of the initial research questions to the drafting of the focus group protocol, my positionality as a White man studying issues of race remained at the forefront of my mind*.’ Applying reflexivity, Allan (2022, 7) stated, ‘*I think my failure to recognise my whiteness was shaped partly by the dynamics of the research process*.’

Bourke (2014, 4) on reflexivity believes that ‘Interpretation consists of two related concepts: the ways in which the researcher accounts for the experiences of the subjects and of her or himself, and the ways in which study participants make meaning of their experiences. Related to subjectivity is the expression of voice that results in the reporting of research findings. Through this voice, the researcher leaves her or his own signature on the project, resulting from using the self as the research instrument and her or his subjectivity.’

Engaging reflexivity in practice led Allan to state that ‘*this was the beginning of my understanding of whiteness and white racialised identities in nursing and nursing research*.’ The full pulse and relevance of reflexivity in practice are felt in Allan, (2022, 8) conclusion that:

‘As a nurse, nurse teacher and, more recently, nurse researcher, I was racialised into racist practices throughout my career. I played a part in racialising students… Reflecting back on my research career, it is ironic that I spent a significant amount of time as a researcher investigating racism in the NHS against IENs while ignoring my own positioning as a white researcher and my own white privilege. I interpret my failure to understand my … position in a white supremacist society as a defence against the pain of recognising my … racism or white fragility. I suggest that as a profession, at least in the UK, nurses have some way to go to do this collectively; to really understand the dynamics of racism in practice, education and research.’

It is clear from Allan's account that her reflexivity involves a deep reflection and an appreciation of her positionality as a white researcher benefitting from white privilege in researching IENs. The perceived benefit of white privilege worked in the opposite with Bourke, (2014, 7) conduct of focus group study. According to Bourke ‘*I might have taken my positionality for granted in attempting to engage White students in discussions of race. What can be an explanation for their reticence and refusal to speak about issues of race? One potential answer is that White students might have perceived me as challenging their White privilege. If this were the case, this perception might have eliminated any trust that would have been based on our shared sameness*.’

An IPA researcher must work out and come to know his ‘fore‐having, fore‐sight, and fore‐conception’ before entering a ‘circle of understanding that interprets while investigating the meaning of being of an entity’ (Heidegger, 1962 cited in Converse 2012). For example, in the integration of IEN into the British healthcare study, whereas the lead researcher readily found citations to support the findings of the perceptions from two streams of the interviewees, the lead researcher could not find citations from the dominant literature to support the novel perspective of one‐third of the interviewees in the *IEN* study. This supports Allan (2022) perspective that because nursing is white, there was no formal discussion of racism or ethnicity in nursing. The lead researcher had to employ the reflective journal using reflexivity to understand and discuss the findings of the perception of this stream of the interviewees lived experience on integration. Reflexivity, assisted the lead researcher to compare the novel perspective with the backlash following England loss to Italy in the EURO 2020 football event. Using reflexivity, the lead researcher was able to link the perspective of a stream in the IEN research finding that integration was a ‘*farce’ and non–existent phenomenon*—‘*more a grammatical phenomenon rather than a reality*,’ (Ugiagbe 2022). The perspective of this group resonated with, Mesut Ozil, a retired German Footballer of Turkish ancestry conclusion that even if he (“*Africans”*) spent over 100 years in Germany (“*UK”*), people would still query his (“their”) origin by asking, ‘*where are you originally from*?’. Reflexively, the experiences of the patriotic BME England players following the EURO 2020 Football event, support the idea of differential racialisation by whites of different groups at different times. The storytelling component of Critical Race Theory lends credence to a stream of the respondents as a nondominant group with distinct and unique histories and experiences with oppression and racism (Ugiagbe 2022). This may have informed their unique perspectives, explication of integration in the UK and UK healthcare, and the social assumptions that racism is enshrined in the UK social structures (Ugiagbe et al. 2022). As an ethnic minority researcher, the lead researcher's ability to think, and reflect as researcher in own mother tongue and in the English language was both an advantage and a disadvantage. The constant use of reflexivity and the awareness of the third hermeneutic level (Smith, Flower, and Larkin 2022) in the IPA study contributed to make the lead researcher overcome the complexity from possible clash of values and interpretation of the phenomenon. The lead researcher had to consistently imagine the reader's interpretations being ‘*positioned as attempting to make sense of the researcher trying to make sense of the participant's experience’* (Smith, Flower, and Larkin 2022, 182).

## 7 Conclusion

Using IPA as a methodological approach provides the researcher with an opportunity to a better position to approach the study ‘honestly and openly’ (Smith, Flower, and Larkin 2012, 27). A researcher's cultural competence contributes to shape the entire research process to ensure cross‐cultural validity and reliability (Archibong and Darr 2010). IPA as a methodological approach recognises the personal and professional influence of a researcher's experiences on the research process (Smith, Flower, and Larkin 2012). This is where the appropriate use of reflexivity by the researcher and declaration of a researcher's positionality helps in the third hermeneutic circle examination of the researcher's position, possible bias in the decisions, and the study's rigour, and supports the parable that *… it is the objective onlookers that can judge correctly if a dancer is a good dancer*.

## Ethics Statement

The Middlesex University Research Ethics Committee granted ethical approval for the study on integrating IEN into UK healthcare (application number 2442). Individuals participating in the study were not physically or psychologically harmed. Every legal requirement concerning health and safety regulations, data protection, confidentiality and anonymity, and signed consent to participate were taken into due consideration.

## Conflicts of Interest

The authors declare no conflicts of interest.

## Data Availability

Data sharing does not apply to this article as no datasets were generated or analysed during the current study.
